# Collective trauma in northern Sri Lanka: a qualitative psychosocial-ecological study

**DOI:** 10.1186/1752-4458-1-5

**Published:** 2007-10-04

**Authors:** Daya Somasundaram

**Affiliations:** 1 Department of Psychiatry, University of Adelaide, Australia; 2 Scholar Rescue Fund, Institute of International Education, New York, USA; 3 University of Jaffna, Sri Lanka

## Abstract

**Background:**

Complex situations that follow war and natural disasters have a psychosocial impact on not only the individual but also on the family, community and society. Just as the mental health effects on the individual psyche can result in non pathological distress as well as a variety of psychiatric disorders; massive and widespread trauma and loss can impact on family and social processes causing changes at the family, community and societal levels.

**Method:**

This qualitative, ecological study is a naturalistic, psychosocial ethnography in Northern Sri Lanka, while actively involved in psychosocial and community mental health programmes among the Tamil community. Participatory observation, key informant interviews and focus group discussion with community level relief and rehabilitation workers and government and non-governmental officials were used to gather data. The effects on the community of the chronic, man-made disaster, war, in Northern Sri Lanka were compared with the contexts found before the war and after the tsunami.

**Results:**

Fundamental changes in the functioning of the family and the community were observed. While the changes after the tsunami were not so prominent, the chronic war situation caused more fundamental social transformations. At the family level, the dynamics of single parent families, lack of trust among members, and changes in significant relationships, and child rearing practices were seen. Communities tended to be more dependent, passive, silent, without leadership, mistrustful, and suspicious. Additional adverse effects included the breakdown in traditional structures, institutions and familiar ways of life, and deterioration in social norms and ethics. A variety of community level interventions were tried.

**Conclusion:**

Exposure to conflict, war and disaster situations impact on fundamental family and community dynamics resulting in changes at a collective level. Relief, rehabilitation and development programmes to be effective will need to address the problem of collective trauma, particularly using integrated multi-level approaches.

## Background

Disasters, whether natural or man made, are now well known to cause a variety of psychological and psychiatric sequelae. These could range from adaptive and constructive coping responses in the face of catastrophic events to understandable non-pathological distress as well as a number of recognizable psychiatric disorders. Conditions like Acute Stress Reaction (ASR, the old disaster syndrome), Posttraumatic Stress Disorder (PTSD), depression, anxiety, somatoform disorders, alcohol and drug abuse [1,2], and in the long term, complex PTSD [3], enduring personality changes or Disorders of Extreme Stress Not Otherwise Specified (DESNOS) have been shown to occur after disasters [4]. Evidence based and effective, modern treatments like Cognitive Behaviour Therapy (CBT) and pharmacotherapy for PTSD to help individuals affected by the trauma of disasters to recover are now available in western countries [5].

However, there is less recognition or understanding of the effects disasters have at the supra-individual levels as well as about appropriate interventions at these levels. There are many reasons for this relative deficiency. First, the field of disaster studies is itself rather recent. For example the diagnosis of PTSD was accepted only in 1980 with the American DSM III [6].

Secondly, modern psychology and psychiatry have had a western medical illness model perspective that is primarily individualistic in orientation[7]. Geertz describes the Western concept of self as "...*a bounded, unique, more or less integrated motivational and cognitive universe, a dynamic centre of awareness, emotion, judgement, and action organized into a distinctive whole and set contrastively both against other such wholes and against its social and cultural background....is a peculiar idea within the context of world cultures*" [8]. The 'Kantian concept of an autonomous self' [9] and '*Enlightenment values of individualism*' [10] has come to mould western ways of experiencing the self, the world and events. PTSD is clearly a condition that exclusively afflicts the individual self, the traumatic event impacting on the individual psyche to produce the PTSD. However, it is being increasingly recognized generally that we need to go beyond to the family, group, village, community and social levels if we are to more fully understand what is going on in the individual, whether it be his/her development, behaviour, emotion, cognition or responses to stress and trauma as well as design effective interventions to help in the recovery and rehabilitation of not only the affected individuals but also their families and community. For when the family and/or community regained their healthy functioning, there was often improvement in the individual member's wellbeing as well. The sense of community appears to be a vital protective factor for the individual and their families and important in their recovery.

This broader, holistic perspective becomes paramount in non-western, 'collectivist' cultures which have traditionally been family and community oriented, the individual tending to become submerged in the wider concerns [7,11]. Collective events and consequences may have more significance in collectivistic communities than in individualistic societies like the US and Australia. It may also be important to bear in mind that societies are in flux, changing. With modernization and globalization, collectivistic societies are also increasingly becoming individualistic and consumer oriented. There may also be traditional subcultures within the bigger, individualistic culture. In collectivist societies, The individual becomes embedded within the family and community so much so that traumatic events are experienced through the larger unit and the impact will also manifest at that level. The family and community are part of the self, their identity and consciousness. The demarcation or boundary between the individual self and the outside becomes blurred. For example Tamil families, due to close and strong bonds and cohesiveness in nuclear and extended families, tend to function and respond to external threat or trauma as a unit rather than as individual members. They share the experience and perceive the event in a particular way. During times of traumatic experiences, the family will come together with solidarity to face the threat as a unit and provide mutual support and protection. In time the family will act to define and interpret the traumatic event, give it structure and assign a common meaning, as well as evolve strategies to cope with the stress. Thus it may be more appropriate to talk in terms of family dynamics rather than of individual personalities. There maybe some individual variation in manifestation, depending on their responsibilities and roles within the family and personal characteristics, while some may become the scapegoat in the family dynamics that ensues (see family case histories [12]). Similarly, in the Tamil communities, the village and its people, way of life and environment provided organic roots, a sustaining support system, nourishing environment and network of relationships. The village traditions, structures and institutions were the foundations and framework for their daily life. In the Tamil tradition, a person's identity was defined to a large extent by their village or *uur *of origin [13]. Their *uur *more or less placed the person in a particular socio-cultural matrix.

A word of caution is necessary in trying to romanticize or idealize the family, neighbourhood, village, collective and community which in reality are vague, amorphous terms, and which include within it considerable variation among members as well as negative dynamics like scapegoating, marginalization, exclusion, ostracism and hegemonic tendencies. It would also prove very difficult to define community and collective very precisely, as the borders will invariably breakdown [14]. However for this paper, the discussion addresses cataclysmic forces impacting on these structures and as such consideration of some of these important internal difficulties can be temporarily postponed till the overarching issues are clearer.

A better understanding of the supraindividual reality can be sought through the ecological model of Bronfenbrenner [15] with the micro, meso, exo and macro systems or the individual nested in the family nested in the community [16,17]. The Bronfenbrenner model fits the WHO definition of health which also emphasises the need to look beyond the micro or individual level (see Table 1):

**Table 1:** Dimensions of health

**Dimensions of Health**	**Causes**	**Symptoms**	**Diagnosis**	**Interventions**
Physical	Physical injury Infections Epidemics	Pain, fever, Somatization	Physical illness, Psychosomatic, Somatoform disorders	Drugs treatment, Physiotherapy, Relaxation techniques, massage
Psychological	Shock Stress Fear-Terror Loss Trauma	Tension, fear, sadness, learned helplessness	ASR, PTSD, Anxiety, Depression, Alcohol & Drug abuse	Psychological First aid, Psychotherapy, Counselling, Relaxation techniques, CBT
Family	Death Separation Disability	Vacuum Disharmony Violence	Family Pathology, Scapegoating	Family Therapy Marital Therapy Family Support
Social	Unemployment, Poverty, war	conflict, suicidal ideation, anomie, alienation, loss of communality	Parasuicide, Suicide, Violence, collective trauma	Group Therapy, Rehabilitation, community mobilization, Social Engineering
Spiritual	Misfortune, bad period, spirits, angry gods, evil spells, Karma	Despair, Demoralization, Loss of belief, Loss of hope	Possession	Logotherapy, rituals, traditional healing, Meditation, Contemplation, Mindfulness

"*Health is a state of complete physical, mental, (family), social and (spiritual) well-being, and not merely an absence of disease or infirmity*".

- World Health Organisation (WHO)

The family unit has been included as it is paramount in most parts of the traditional world. When the family is affected, the members too are affected, while if the family is healthy the individual is either healthy or recovers within the family setting. The spiritual dimension has been put forward at various WHO fora but has not been formally accepted yet.

**Figure 1:**
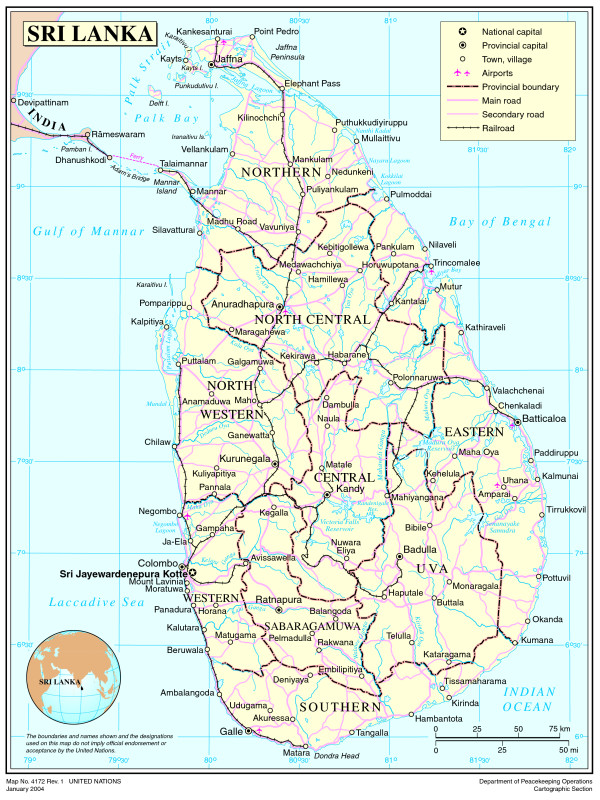
Map of Sri Lanka (from UN-OHCHR).

This reflexive study grew out of the experience of working in disaster, post-disaster contexts: the man made disaster of war and the 2004 Asian Tsunami in Northern Sri Lanka (see Fig. 1). The phenomena of collective trauma first became very obvious to the author when working in the post war recovery and rehabilitation context in Cambodia [18]. During the Khmer Rouge regime, all social structures, institutions, family, educational and religious orders were razed to 'ground zero' deliberately (so as to rebuild a just society anew!) [19]. Mistrust and suspicion arose among family members as children were made report on their parents. The essential unity, trust and security within the family system, the basic unit of society, was broken. Similar changes at the family and community levels became discernable in the Northern Sri Lanka as the conflict continued.

From a conservative society caught up in the world wide modernization and globalization, the minority Tamil society in Sri Lanka became increasingly embroideled in a civil, ethnic war from 1983 onwards. Developing as a defiant reaction to increasing majority Sinhala dominated state policies of discrimination; the youth rebellion took an increasing violent form in opposition to increasing state oppression. The North and East of the country, home to the minority Tamils, bore the brunt of the chronic violence that followed [12]. The state, including the Indian state briefly in the late 1980's, various paramilitaries and Tamil militants have been involved in in cycles of violence, counter violence, terror and counter terror [20]. During a lengthy period of ceasefire, the island was struck by the Asian Tsunami in December, 2004 that affected over 200, 000 people again mainly in the coastal communities of the North and East but also the South. The author was put in charge of addressing the psychosocial needs in the North (see Fig. 1).

The impact of catastrophic events on the individual has been well established internationally [6,21] and was quite clear in Northern Sri Lanka [12]. There have been some observations on the family level too in Northern Sri Lanka [22]. However, it was when it came to addressing mental health problems that the impact on the community became evident. Simple interventions at the individual level were not sufficient. The problems at the community level too had to be understood and addressed if the individuals were to be helped. Further, families and communities had to recover if any meaningful socio-economic rehabilitation programmes were to succeed. In fact, in time most long-term programmes, as in other post disaster settings arond the world [23-25], began to include a community based psychosocial component within the larger socio-economic rehabilitation and reconstruction efforts, [26,27].

In these complex (post) disaster situations, the multi-level WHO definition of health is useful in conceptualizing the causes, effects and interventions for psychosocial and mental health problems found in these devastated communities (Table 1). In reality, these dimensions are interacting systems which are interrelated, each level having effects at other levels so that a holistic approach that integrates all these levels becomes necessary.

For example, physical illnesses like injuries, epidemics and malnutrition resulting from war conditions [28,29], will have direct physical causes, physical symptoms and physical treatment. But it could also have psychological, social and spiritual causes, symptoms and treatment. Physical diseases, in addition to the familiar physical signs & symptoms, also manifest with psychological, social and spiritual symptomatology. At the same time, psychological factors can cause or contribute to physical diseases like Bronchial Asthma, Hypertension, Eczema, and Colitis. Mental illnesses can manifest with physical symptoms such as the common Somatoform Disorders or Somatization. A global perspective shows that mental health problems the world over produces a major proportion of the Global Burden of Disease (GBD), including death, disability and injury due to behavior related problems such as adverse life style, alcohol and drug use, road traffic accidents, war and violence, exploitation, and AIDS [30]. However, the contribution to GBD from disasters is yet to be mapped out [28,29]. Physical and mental illness have social repercussions. Epidemics of physical illnesses like the current HIV/AIDS pandemic has severe socioeconomic implications nationally and internationally. Mental illnesses can cause problems in the family, community and work place due to irritability, paranoia, relationship conflicts, alcoholism and/or drug abuse, domestic violence, morbid jealousy and social withdrawal. In a like manner, social problems like unemployment, poverty, war, and displacement can cause psychosomatic diseases, mental illnesses like depression and suicide [31]. When considering treatment one can also have interventions at all these levels, either alone or in combination. Thus pharmacotherapy or treatment with drugs is the prototype of physical treatment. Psychotherapy, counselling, behavioural and cognitive therapies are some common psychological forms of treatment. While marital, family and group therapies as well as rehabilitation, NGO networking, occupational therapy and vocational training can be considered social forms of therapy. Likewise, it is said that spiritual meaning, hope and strengths will produce resilience and improvement at all the above levels. Viktor Frankl [32] pioneered this form of treatment which he called *logotherapy *after surviving the Nazi concentration camps during World War II.

Previous workers had already drawn attention to the community level problems caused by disasters. Kai Erikson [33,34] gives a graphic account of ***Collective Trauma ***as '***loss of communality***' following the Buffalo Creek disaster in the US. He and colleagues described the 'broken cultures' in North American Indians and 'destruction of the entire fabric of their culture' due to the forced displacements and dispossession from traditional lands into reservations, separations, massacres, loss of their way of life, relationships and spiritual beliefs [35]. Similar tearing of the 'social fabric' has been described in Australian aboriginal populations [36]:

"...*it implies things have to be woven together properly for strength, what a shame our fabric was torn to shreds through invasion, what we have left now is in tatters, repairing fabric can make it weak or sometimes stronger depending on how it is done. It is important to repair the holes and not just cover them over so that when some tension is applied it doesn't fall apart. What kept our fabric strong was spirituality, the invisible thread that binds us all*"

The National Strategic Framework for Aboriginal and Torres Strait Islander Health [37] states:

"*The sense of grief and loss experienced by generations of Aboriginal and Torres Strait Islander peoples in relation to dispossession, to the disruption of culture, family and community and to the legislated removal of children has contributed to **ongoing problems in emotional, spiritual, cultural and social well-being **for Aboriginal and Torres Strait Islander individuals, families and communities*."

O'Donoghue [38] describes the collective trauma,

"Aboriginal *culture has been subjected to the most profound shocks and changes. It is a history of brutality and bloodshed. The assault on Aboriginal people includes massacres, diseases, dispossession and dispersal from the land... I cannot overstate the **traumatic consequences **of policy and the **destruction of **Aboriginal and **community life **that resulted*."

Nadew [39] in his survey of psychosocial and mental health problems among the aboriginal population found a very high prevalence of conditions like PTSD (55 %), depression (22%), alcohol abuse (73 %), and violence. He linked it to the long-term and massive trauma suffered by the aboriginal population. O'Shane identifies the loss of pride, identity, self respect, language, songs, laughter, spirituality, relationships, traditional knowledge and skills in the group as a whole [40]. The consequences are trans-generational, being passed onto later generations as was found with World War II holocaust survivors [41].

The high incidence of mental health problems, alcohol and drug abuse, physical and sexual violence, child abuse and family disharmony found among indigenous populations around the world can be the result of the break up of traditional culture, way of life and belief systems. Instead of the usual response of incarceration, exclusion and suppressive measures, significantly, there are now attempts to repair the torn social fabric. The Dulwich centre in Adelaide has used narrative therapy to 'reclaim community' [42]. By sharing stories at community gatherings relationships, connection and links are re-established, traditional values, beliefs, knowledge, skills and hope are re-kindled, giving rise to community solidarity and support. The method has been expanded to other indigenous populations around the world.

There was a description of '***cultural bereavement***' due to the loss of cultural traditions and rituals in Indochinese refugees in the US [43] and collective trauma due to the chronic effects of war [44]. More recently, a number of discerning workers in the field have been drawing attention to the importance of looking at the family [26,27,45,46] and cultural dimension [7,45,47-49] following disasters. Finally, Abramowitz [50] has given a moving picture of '***collective trauma***' in six Guinean communities exposed to war.

Borrowing from the individual psychopathological descriptions, the term collective trauma is being introduced in this study in a metaphorical sense to represent the negative impact at the collective level, that is on the social processes, networks, relationships, institutions, functions, dynamics, practices, capital and resources; to the wounding and injury to the social fabric. The long lasting impact at the collective level or some have called it tearing in the social fabric would then result in the social transformation [51], of a sociopathic nature that can be called collective trauma. This study attempts to describe the phenomena of collective trauma, to delineate the symptoms and community level interventions that can be used in such contexts.

## Methods

This qualitative, ecological study is a naturalistic, psychosocial ethnography [52] in two contexts: The main focus is on the ongoing chronic civil war situation from 1983 onwards contrasted to the pre-war conditions and the post Dec. 2004 Tsunami recovery effort in Northern Sri Lanka. The ecological study follows Bronfenbrenner [11]:

"...*an effort to investigate the progressive accommodation between the growing human organism and its environment through systematic contrast between two or more environmental systems or their structural components, with a careful attempt to control other sources of influence either by random assignment (planned experiment) or by matching (natural experiment).... There are instances in which a design exploiting an experiment of nature proves a more critical contrast, insures greater objectivity, and permits more precise and theoretically significant inferences- in short, is more elegant and constitutes "harder" science- than the best possible contrived experiment addressed to the same research question*."

Participant Observation, in depth case studies [53], key informant interviews and focus group discussions as well as several quantitative, individualistic psychosocial and mental health surveys published elsewhere [12,54,55] provided the data for the study. The focus group discussions have included groups from the community, village, local government (G.S.-village headman, teachers, social workers, priests); displaced camp and relief workers; District (GA-District Authority, Non-Governmental Organization (NGO), militant) committees; National (Health Ministry, Presidential Task force); and International NGO (INGO's, UN) groups that discussed and debated mental health and psychosocial issues. The author had the unique experience of working as a mental health professional in these settings. Though being an Asian, ethnic Tamil, the author had most of his education and professional training abroad, particularly in the west, providing both an 'insiders' and 'outsiders' view point.

The main purpose of this study is an attempt to phenomenologically describe and understand the familial and societal factors involved so as to better design and implement more effective, appropriate and workable interventions, policies and programmes in a post-disaster context. In addition there is a plea for prevention of such disasters and the consequences they entail by documenting and describing the devastating effects of war and disasters on families and communities.

## Results and Discussion

### Individual

Several surveys of individual level trauma and its effects in the contexts of war and post tsunami in North Sri Lanka have shown widespread traumatization and considerable psychosocial sequelae in the different population groups [12,54,55]. In depth case studies show the variety of psychopathological responses [53]. An epidemiological survey using the UCLA PTSD Child Reaction Index with expert validation (Kappa .80) [56] carried out in the Vanni, an area in the North of Sri Lanka, found that 92% of primary school children had been exposed to potentially terrorizing experiences including combat, shelling, and witnessing the death of loved ones. In 57% of the children, the effects of these experiences were interfering considerably with their daily life (e.g., social withdrawal and weakening school performance). About 25% were found to suffer from PTSD. Our epidemiological survey of school children in the North and East found that 47% of those who had been exposed directly to the tsunami and 15% of children not exposed had PTSD (all living in a war context).

Apart for the individual suffering and disability, such a large prevalence of psychiatric disorders in a population would lead to a cumulative effect that would cause considerable social pathology and dysfunction. However, in this abnormal situation many of the reactions, which would be considered pathological in normal times, would become the norm – a normal reaction to an abnormal situation. For example, startle reactions to sudden loud noises like backfiring of motorcycles, banging doors or firecrackers and nightmares of war events, which are characteristic symptoms of PTSD, were widely prevalent and not considered abnormal. Thus many with so-called diagnosable psychiatric disorder like those who would be identified in population surveys may not seek help, at least western psychiatric help. This is clearly shown in the low percentage of PTSD and similar post trauma syndromes in psychiatric out patient services (approximately 5%). Some were seeking help through other avenues such as the traditional sector or general health care facilities with somatic complaints [57] or more traditional idioms of distress like *Perumuchu *(Deep sighing breathing signifying worries and emotional burdens) in the Tamil community. An important contentious issue is whether those diagnosed as having psychiatric disorder using western categories are in fact having a mental illness, the categorical fallacy [58]. The person, his/her family and community often did not recognize or accept a psychiatric disorder.

**Figure 2:**
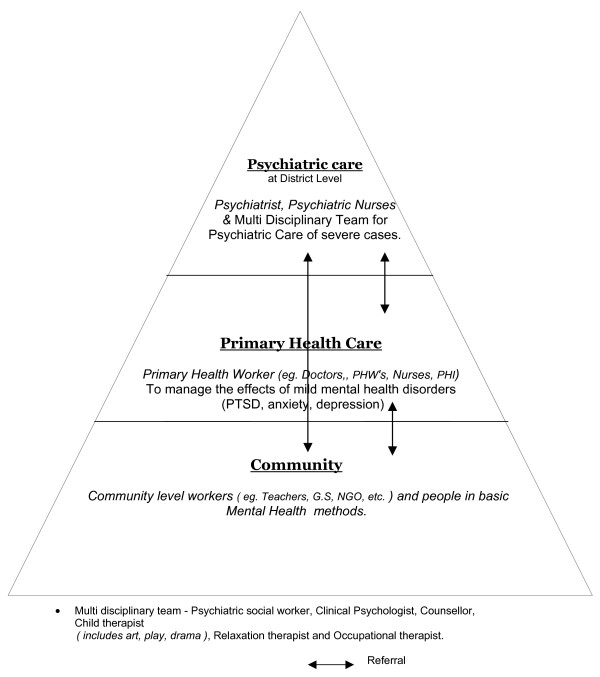
Referral structure for management of mental health problems (at District Level).

A further problem is how effective or appropriate are western treatments such as psychopharmacotherapy or state of the art CBT even in those with manifest dysfunction. In Northern Sri Lanka, CBT was not possible as there were no clinical psychologists. Even the recommended psychopharmacological agents in the west, SSRI's, were not available (In the post tsunami period these treatments did become briefly available thanks to the international goodwill). Our experience in Northern Sri Lanka in these different contexts has been that a small minority with more severe dysfunction do benefit from psychiatric treatment which could also include cultural techniques like yogic relaxation methods [59]. When individuals improved it also helped their families, communities or refugee camps where they lived. In addition, the conventional psychotic illnesses like Schizophrenia and Bipolar disorders needed continued medical treatment. However, due to the disasters much of the health infrastructure and resources were destroyed or depleted and did not function adequately. Mental health services using essential psychotropic medication, out reach clinics in the periphery and badly affected areas, and training of primary health care workers enabled minimum cover. The generous post tsunami international support facilitated the introduction of decentralized mental health services at the district level (Fig. 2). Indeed, community mental health programmes that do not include the possibility of addressing the problems of those with severe mental disorders would fail in the eyes of the community and cause a breakdown in the smooth functioning of the setting where they were. Nevertheless, it was not feasible to treat the large numbers affected with minor mental health problems due to the disasters with western psychiatric treatment. Rather, a community based programme to rebuild the damaged family units and social structures, networks, resources and relationships encourage re-establishment of helpful traditional healing rituals and practices; group meetings and functions, in short to start the community working again appeared to be more judicious. Training a variety of grass root workers in basic mental health and psychosocial skills was the most effective way to accomplish this [60] When the family and community regained their function, individuals recovered their confidence, motivation, capacity and skills.

### Family

The war and the tsunami had a major impact on the functioning family system. From the loss of one or both parents, separations and traumatization in one member, pathological family dynamics adversely affected individual family members, particularly the children.

The traditional family unit as the basic social institution has barely survived but its function has been irrevocably changed by the chronic conflict. The cohesiveness and traditional relationships are no longer the same. Compared to before the war, it is a common complaint that children no longer respect or listen to their elders, including teachers. These changes, attitudes and perceptions, like many others, may have antedated the war but could have been accelerated by it. A seniour British INGO (SCF) worker who had served in Jaffna for a long period made the observation of the common day to day occurrence at the pervasive check points in the North and East. Tamil parents quickly change their behaviour and tone (in contrast to what the child has seen at home or elsewhere) when dealing with the security forces. They, perhaps unconsciously and with the best of intentions (to safeguard their children and to avoid unnecessary hassle), assume a submissive posture (removal of hat, bent head and body, low and almost pleading tone of voice, pleasing manner with a smile) when accosted by the security forces (e.g. at check points). The children will observe this change without comprehending the full purpose (perhaps to avoid the child being detained), comparing it to their demeanour at home and in time loose faith in his or her parents. A strong influence has been the contemptuous way elders and community leaders have been treated by the authorities and the submissive way they have responded. Elders are perceived as being powerless and incompetent in dealing with war and its consequences, a point often made by the young militants. Parents were careful about what they discussed in front of their children as the child could inadvertently let this out in school or during play, particularly if it was something against the Tamil militants. Elders have also been traumatized by the war, affecting their functioning, relationships and parenting skills.

Due to the peculiarities of the war, males were more often targeted and were at high risk to be killed, arrested, detained, disappeared, join the militancy or to migrate. For example in the small fishing village of Chavatkaddu, where we set up a widow's group, there were 180 widows, many of whose husbands were killed at sea, a highly contested area between the Sri Lanka Navy and the Tamil militants. Altogether there are 19,090 female headed households in Jaffna. A great number are widows who have lost their husbands due to the war. The effect on the family, the widow [61], and the children has been immense. The loss of one member of the household, particularly the breadwinner has a marked impact on the family dynamics. Absence of members of the family due to death, injury or displacement will create immense gaps in the functioning of the family unit. The uncertainty or grief about the missing member will add to the maladaptive family dynamics that will ensue. The loss of the essential unifying role of the missing member can cause disruption and disharmony within the family. A common situation is where the father has been detained, 'disappeared' or killed but the family members are not sure of his fate. They are caught in a '*conspiracy of silence*' where further inquiries may lead to more problems for the father were he still alive and the mother may not be able to share the truth with the child. The child then presents with behavioural problems. The mother has to adapt to all the negative implications of being a 'widow' in this society. The role of the mother has undergone momentous change with increasing non-traditional responsibilities, activities and "liberation" [62].

A more tragic situation happens when the disappearing is done by a local Tamil militant group. Here the conspiracy of silence is much deeper. A disappearance by the army or security forces is acceptable within the community. But disappearance by the Tamil militants is something the widow and her children cannot talk about even in the community or inner family circle. Apart from the danger and risk of repercussions, the disappeared is made into an undesirable, a traitor. The family itself often becomes ostracized by society. The widow cannot express her emotions at all, even to herself; the deception thus goes much deeper into oneself. In a case that I was seeing, the widow was suppressing the memory of her husband and in time he disappeared from her consciousness as well as from his children and society.

In 1996 there were reported to have been 600 disappearances in Jaffna [63] and in the current period from Dec. 2005, there have already been 1300 Island wide [64]. A study by Shantiaham [65] involved the follow-up of the families of the disappeared, assessment of needs and psychosocial support. When entering the homes of the disappeared, the atmosphere was akin to a funeral house, even years after the disappearance. The mildest of conversations linked to the disappeared, would set off tears and crying. The house was not lively, it was quiet and moody. In the case of the disappeared there is no closure of the death, no certainty about the fate of the person. It would be disloyal to even consider that the person could have been killed.

A study assessed the impact of displacement in the North on functioning of the family system [22]. Psychological disturbances particularly depressive symptoms were much more common in displaced families than in those living in their own homes. In displaced children, separation anxiety was common as were cognitive impairment, conduct disorders and sleep disturbances. Disturbances in family dynamics particularly disputes and quarrelling between father and mother were attributed to economic stress, lack of privacy and interference of others in over crowded camps. Other war related trauma like torture and loss of a limb due to landmine injury had a direct impact on families [66]. The loss of a limb led to feelings of inferiority and shame that made family life difficult. In some cases, husbands left their wives. Torture survivors who returned to their families often were not able to function as before. They were socially withdrawn, had difficulties with intimate relationships, they were irritable and not motivated to work or be active [67]. These situations changed the family dynamics. In one case referred to the psychiatric unit, there was a role reversal with the wife going to work and the husband trying to cope with household chores. Due to his feelings of inadequacy, he attempted suicide. He had clear PTSD symptoms, yet the family refused to accept mental health help. When a family member develops a psychiatric disorder like PTSD, depression or substance abuse due to traumatization, the symptoms and social dysfunction had an adverse effect on the family as well.

In the case of the tsunami, there were more women who died compared to men as elsewhere [68]. Perhaps the women were less capable of surviving, not having the skills in the sea. Women may also have been home when the tsunami struck. This left many male widows who found it difficult to cope with the remaining families, not having the skills to look after children, prepare food or do routine household chores. Some took to alcoholism. Suicidal ideation, attempts and suicide was reported to be high. In time some re-married creating problems for the children. Often the children were given to other relations to look after. Cases of child abuse were reported in some of these arrangements. The attempts to adopt children who lost one or both parents or to put them in institutions were resisted by child protection authorities. Initially there had been reports of abduction of orphans, so called tsunami babies as well as interest from people abroad to adopt tsunami orphans. Thus in many ways the impact of the tsunami on the family was unique.

### Community

Disasters have an effect not only on individuals [12], but also on their family, extended family, group, community, village and wider society. During civil conflicts, arbitrary detention, torture, massacres, extrajudicial killings, disappearances, rape, forced displacements, bombing and shelling became common (see Tables 2).

**Table 2:** Distribution of war stress in the community

**Stress factors**	**Community [44] (n = 98)**	**OPD^c ^[47] (n = 65)**
**Direct stress**		
Death of friend/relation	50%	46%
Loss to property	46%	55%
Injury to friend/relation	39%	48%
Experience of bombing/shelling/gunfire	37%	29%
Witness violence	26%	36%
Detention	15%	26%
Injury to body	10%	9%
Assault	10%	23%
Torture	1%	8%
**Indirect stress**		
Economic difficulties	78%	85%
Displacement^a^	70%	69%
Lack of food	56%	68%
Unemployment	45%	55%
Ill health^b^	14%	29%

^a^Before the 1995 mass displacement when the figure would have reached almost 100%

^b^Ill health due to war related injuries including amputations due to landmine blasts, epidemics like malaria, reduced resistance to infections (due to stress and malnutrition), septicemia etc. had debilitating mental effects.

^c^Out Patient Department (OPD) at General Hospital, Jaffna

Whole communities or villages were targeted for total destruction, including their way of life and their environment. According to a Save the Children, UK publication [69] on the nature of current conflicts:

"*Civilians are no longer 'incidental' casualties but the direct targets of violence*.*Mass terror becomes a deliberate strategy. Destruction of schools, houses, religious buildings, fields and crops as well as torture, rape and interment, become commonplace. Modern warfare is concerned not only to destroy life, but also ways of life. It targets social and cultural institutions and deliberately aims to undermine the means whereby people endure and recover from the suffering of war*....."

"*A key element of modern political violence is the creation of states of terror to penetrate the entire fabric of economic, sociocultural and political relations as a means of social control" *[70].

It maybe more accurate to say that the nature of war has changed. Instead of the old fashioned wars between states for control of territory, where sides will fight each other on battlefields till one emerges as a victor, modern wars have now become internal, civil wars, where the conflict is more psychological for control of loyalties through intimidation and terror, the fighting occurs within civilian populations, where 90% of casualties are civilians [71]. Apart from wars for complete extermination, that is genocide, the goal of modern warfare is more for absorption and assimilation in to one dominant culture and way of life. The minority is expected to forgo its own culture and identity and merge with the or become subservient to the dominant culture. Whey they try to resist the process ethnic or civil conflict erupts.

In this way, the civil war in Sri Lanka began as an ethnic confrontation between the majority Sinhalese and Tamil minorities, where the majority sought to impose their language and religion. The resultant conflict has had a profound impact on Tamil village traditions, structures and institutions that had been the foundations and framework for the their daily life causing fundamental, irrevocable change in these processes [72,73]. Good examples are the systemic attacks on all the Tamil villages in the Trincomalee District which eventually displaced all of them into the city or to other districts. Another example is the LTTE's forced eviction in the early 1990's of the Moslems of the North with 48 hours notice, many of whom still languish in refugee camps in the South.

Apart from direct attacks, whole villages of all three communities, Sinhala, Tamil and Muslim, have been disrupted, displaced and uprooted due to the ongoing conflict. Examples are the fishing community and farmers: From the beginning, for alleged security reasons, fishing in the North and East has been restricted. This has included bans on fishing in large areas, restrictions on the distance where fishing can be done (usually restricted to short distances offshore). Whole fishing communities have been displaced, such as from Myeliddy, a fishing village on the northern coast, from early 1990's. The displaced families from these communities can be found dispersed in a number of refugee camps or where several families occupy makeshift accommodation in abandoned homes. Inter and intra family conflict is rife with the once active fishing folk observed to be despondent and hopeless. Fishermen are at increased risk of death, disappearance, detention, and injury due to the war in the sea. As a result the highest number of widows come from this community. This has had a terrible toll on this community. Many have shifted to other occupations, some are still unemployed living off government rations, others have left the area. They have lost their way of life and culture. Many yearn for the days when they were able to fish freely and lead a fisherman's life in their village. They often report dreams of living in their old homes and going fishing in the sea. Before 1983, fishing was a very fruitful occupation, the catch was good off the long North East coast, and a considerable part of the fish was transported to Colombo and other areas in the Island. In the whole island the North East was a leading area for fishing. The coastal communities were thriving hives of activity.

With the ceasefire in 2002, many of the fishing families returned to their coastal villages and restarted their fishing activities step by step. When the tsunami struck in December 2004, all this was again destroyed. The sea had been a vital and intimate part of their lives. The sea was called the 'mother' in Tamil in their folk songs, narratives, literature and common parlance. When the sea rose up and struck with such destructive furore, a fundamental, organic bond was broken. In the early days after the Tsunami most of the fisherman vowed never to go back to the sea. Many of their songs and discourse of this time expressed this loss, grief and feelings of being abandoned by the mother, rebuked and punished. The slow mending of this organic relationship took time. Eventually most fisherman returned to fishing, families slowly moved closer the sea from where they had initially been displaced and then feared returning to. Community mental health programmes worked on reducing this fear (by various desensitisation techniques) and encouraged the return to fishing. The resumption of conflict has displaced many of these communities again. Significantly, the recent disappearances and extrajudicial killings have targeted the leaders of the fishing organizations who had become effective socio-politically in working for the betterment of their community.

It has been a similar tale with farmers, many of whom have been displaced from their traditional lands, and have lost all their equipment. Some are unable to cultivate their land, as it is located in restricted 'security zones' or is mined. Some have abandoned their traditional occupation as it no longer profitable, given unavailability or cost of agricultural inputs like fertilizers, insecticides and fuel, or lack of access to markets for their product. The ubiquitous presence of buried landmines creates a pervasive apprehension in the back of the minds of people, making them ever vigilant, cautious about walking freely on the land, afraid of putting a wrong foot somewhere. Some developed nightmares of being the victim of an exploding landmine. The once beloved land itself becomes polluted, a source of terror [66].

Other traditional trades like carpenters, masons and bakers have been affected similarly. The Moslems, many of whom were part of a very prosperous business community, or specialized in other occupations like tailoring, tinkering, leather work and commercial entrepreneurship have lost their occupations and way of life due to their forced eviction. They had tried tentatively to return after the 2002 ceasefire but never felt secure or reassured that abductions for ransoms and eviction would not be repeated. With the resumption of hostilities, they have again fled. The Sinhalese were well known bakers in the North and East, but have now all left.

As already mentioned, in these various rural communities, the village or *uur*, was the secure and familiar environment with traditional way of life, supportive social structures, institutions and functioning. With the disasters, both the war and tsunami, many villages have ceased to exist. Due to dislocation villagers have been separated so that the sustaining network of relationships, structures and institutions have been lost. Even when people have returned, the village was not the same. Many were complete newcomers. The old structures and institutions were no longer functioning. Thus the protective environment, the social fabric, provided by the *uur *is no longer there.

Similarly, in the life of Tamils, their home (*veedu*) is very important. There will be a history of the home. The ancestral relations who have died, will continue to have connections with the home. They will be remembered and considered as if they were present in the home, especially when rituals are performed in the home. There is a Tamil tradition of being loyal to the home. If one makes a big mistake, he feels guilty of having betrayed his home. There is a biological link between the home and the people who are dwelling in the home. When one is in the home, or when he or she come from outside into the home or when they are away from the home and think of the home, they feel security and peace (like a baby in the womb). When they area forced to leave the home suddenly with the whole household for long periods, this biological link breaks. This affects the mental condition in several ways. People believe that ghosts or demons will occupy the homes which are left alone, unoccupied for a long time. Repeated displacements, which have affected almost everyone at one time or another, have forced the Tamils to abandon their homes. People who returned to their homes after displacement, felt there was a change in the organic bond. They could not re-establish the relationship with their homes. Many have left their homes in a state of disrepair, occupying a part of the home with makeshift arrangement, ever in the ready with an emergency bag ready to move.

Some catastrophic events were of such a scale that it left an imprint on everyone, on the '*groupmind*', on thinking patterns and memory. It changed the lives of individuals, their families and their communities in fundamental ways, it transformed society [51] and the experience passed on into the collective memory to be recounted in stories, narratives and folklore, songs, poetry and dramas; to influence future generations through subtle social processes, so that it may be appropriate to speak of an impact on the collective unconscious. The mass exodus of 1996 was one such experience [74]. Apart from the forced breaking of the bonds with their homes and village, the trek of over 400,000 people in the middle of night with rain and shells changed everyone. They left in terror and not by choice, with few possessions, roads clogged with crowds moving slowly, step by step, the less able, the elderly, falling by the wayside; and finally arriving in makeshift, inadequate accommodation with very poor facilities or none at all. People lost their identity, pride, dignity and hope.

Other major events having an impact on the collective unconscious were the so-called July 1983 ethnic holocaust; the burning of the Jaffna public library with its irreplaceable old Tamil manuscripts and books (sometimes referred to as cultural genocide); the Indian military operation to capture Jaffna in October 1987 (rupturing a bond with another traditional mother); and the tsunami of December 2004. Some widespread phenomena seen during the war like 'disappearances', torture and landmines, also had a long-term effect on the collective unconscious.

Noteworthy, in our community survey [54] is the finding that 1% of the study population had been tortured, but the figure reached 8% in the OPD patients [57]. Torture was used as a routine procedure carried out on all those detained [67]. It was developed into a physical and psychosocial tool to break the individual personalities of those who tried to resist, as well as an encompassing method to coerce a community into submission. Many individuals did not survive torture, but those who did were released in a broken condition; or when dead, their maimed bodies were conspicuously exhibited to act as a warning to others. It became one aspect of institutionalized violence and laws were passed, such as the Prevention of Terrorism Act and Emergency Regulations, which facilitate prolonged incommunicado detention without charges or trial, in locations at the discretion of the Security Forces, and allowed for the disposal of bodies of victims without judicial inquiry. These legislations legitimized the use of torture and death in custody [75]. Thus torture became institutionalised as an aspect of state terror. It was similarly used by the militants but without the legal veneer. The Istanbul Protocol for the investigation and documentation of torture project team speak of community trauma by the creation of a '*repressive ecology*' based on imminent, pervasive threat, terror and inhibition that causes a state of generalized insecurity, terror and rupture of the social fabric [76].

The chronic climate of terror, insecurity and uncertainty was a prominent cause of the collective trauma due to the war [77], but was not seen with the tsunami. The natural disaster was a one off catastrophic event that left a trail of destruction and loss but did not continue to exert a prolonged effect. As a result the severity of the collective trauma was much less. That war was man made also appeared to add to severity of the traumatic effect. The tsunami on the other hand was attributed to an act of nature or the wrath of God for some wrong doing, karma, often of a collective nature. In N. Sri Lanka, people had been exposed to multiple traumatic events (in N. SL the average was over 6 events) so much so that the condition of chronic traumatic stress maybe a better description [78]. The Tsunami was an additional traumatic event on top of many more. One study found that there was a building block effect, those who had been already traumatized by war were at an increased risk to re-traumatization by the tsunami [55]. Further such indirect effects of disasters (Table 1) like displacement, unemployment, poverty, ill health, malnutrition and socio-economic hardships could be as debilitating and traumatic in its long term effect.

Another ecological factor that was a cause of collective trauma was the breakdown in intersetting communication and knowledge [15] between the mesosystem of the community and the macrosystem of those in authority. Those in authority, the military, militants and the state, held all the power and failed to communicate essential information. Apart from the language barrier between the Sinhala (and briefly Indian) state and the Tamil community, decisions and actions appeared arbitrary and dictatorial. There was no genuine relationships or attempts to communicate except for competing versions, rumours and propaganda exercises through the media that were more confusing to civil society [12]. Accurate and helpful information is considered essential for maintaining and promoting mental health in disaster situations. Access to essential information and awareness programmes are basic mental health strategies. The Tamil militants were also careful not to allow any congenial relationships to build up between the state, the military and civil society. Any positive developments in that direction were dealt with harshly. Public relations exercises were often ill conceived. There were some belated attempts by the security forces to learn the local language, Tamil.

The loss of leadership and the talented, skillful, resourceful persons, the professional, technocrats, and entrepreneurs from the community has had devastating consequences. Many left over the years due to increasing difficulties, traumatic experiences and social pressure from family and colleagues, the so called 'brain drain'. Those who remained have been targeted by those aspiring to rule the community. The various authorities vying for the loyalty and subservience of the community have ruthlessly eliminated what they have perceived as obstruction to their power and control. Apart from the extrajudicial killings of the state and its allied paramilitary forces, the internecine warfare among various Tamil militant organizations competing for the loyalty of the community have resulted in the elimination of many of its own ethnic, more able, civilians- a process of self-destruction, auto genocide. Those with leadership qualities, those willing to challenge and argue, the intellectuals, the dissenters and those with social motivation have been weeded out ('*Pullu Kalaithal'*- those eliminated are labeled as anti social elements or traitors). They have either been intimidated into leaving, killed or made to fall silent. At these shifts in power, recriminations, false accusations, revenge and retribution were very common. It happened in 1987 (IPKF, the Indian intervention); in 1990 (LTTE takeover), in1996 (SLA control) and is currently happening as a free-for-all currently after 2005 with the collapse of the ceasefire.

Bronfenbrenner [15] warns of the destructive consequences to a society which experiences the systematic degrading and debilitation of its richly talented members. This is the loss of vital resources [16], the destruction of social capital, the nodal points of vibrant relationships and essential networks which is a prominent cause of Collective Trauma. Without leadership and organization, vital networks and working relationships have collapsed, leaving the community easy prey to competing propaganda, authoritarian control and suppression. Many have observed that ordinary people in Jaffna have become passive and submissive. These qualities have become part of the socialization process, where children are taught to keep quiet, not to question or challenge, accept the situation, as assertive behaviour carries considerable risk. The creative spirit, the vital capacity to rebuild and recover is being suppressed.

An important ingredient in the social recovery process is resource gain cycles [16]. Unfortunately, after extreme stress the opposite occurs – rapid and turbulent loss cycles [16]. Vicious spirals of loss are set in motion due to cascading patterns of multiple stressors where the loss of one resource triggers other losses [17]. Hobfoll [16] points out that '*major trauma cycles not only spirals (downwards) for the individual in a personal sense with anxiety, depression and loneliness, but also often result in reduced social involvement, diminished interest in life and family feelings of social detachment and a sense of alienation... Moreover, PTSD has a stress-contagion influence producing psychological distress in loved ones*...' For example in the context of war in N.S.L., displaced communities had to first face the traumatic loss of loved ones, their homes, village structures and relationships, witnessing horrifying events before being compelled to flee to perceived safer havens but ending up in crowded, refugee camps with inadequate facilities. Here stressors of unemployment, malnutrition, illness, socio-economic difficulties within an atmosphere of uncertainty and insecurity compound their already precarious plight. The uprooting from the familiar village environment often meant loss of social support networks, traditional leadership, rituals and practices. For community healing and recovery, this vicious cycle has to be broken and a resource gain cycle instituted. Psychosocial and multisectorial interventions will have to address these various problems simultaneously in parallel, in an integrated and holistic way if these negative processes are to be reversed. However, the most effect way to the stop the downward spiral and break the vicious cycle would be to stop the war!

A unique context of the military situation in Jaffna was geographical in that it was a peninsula, connected to mainland Sri Lanka by a narrow isthmus, a thin causeway called Elephant Pass, blocking of which would effectively cut off Jaffna from the rest of Sri Lanka and the world (see Fig. 1). This happened several times during the two decades of war, sometimes lasting for years. Communications including telecommunications and postal services, electricity, transport and travel were all blocked except for very limited air and sea travel. They were attendant shortage of food, fuel, medicine and other essential items. Travel was allowed only after an elaborate pass and permit system. During these times, Jaffna was often under what was perceived as 'foreign' occupation with conspicuous security arrangements consisting of armed guards, weapons of all types, check points, regular patrolling, frequent search operations, arrests, detention, abduction, disappearances, skirmishes, guerilla attacks, ambushes and counter killings by the militants, all this hidden from the eyes of the media, the rest of the island and the world. The atmosphere created a feeling of entrapment, of being besieged. The conditions were compared to being in an 'open prison' by some of the community leaders, the Bishop of Jaffna [79] and Surgeon of the Jaffna Hospital, Dr. Thayalan Ambalavanar [80] among them. An illuminating social experiment of simulating prison conditions were carried out by Zimbardo and colleagues [81]. The power differential between the guards who held and exercised arbitrary control over the prison population and the interpersonal dynamics between the two groups soon manifested in the guards increasing aggressive, brutal, dehumanizing and hostile behaviour. On the other hand the prison population showed a syndrome of passivity, dependence, depression, helplessness, loss of identity and submissiveness. In addition, as seen in real prison systems by powerful group networks within the prisoners, in the Jaffna situation too there was the more pervasive 'counter-control' by the Tamil militants through social pressure, intimidation, killings, abductions and internal terror trapping the civil population between the two forces.

A pernicious element in the collective traumatization of this ethnic war was the systemic nature, the institutionalization of the violence, the terror and counter terror. It became structural, becoming entrenched in the laws of the land, in the way the state treated the minorities, pervading all relationships and activity. Beginning with discrimination and inequity, the emergency and anti-terror legislation, to total militarization of society and targeting of Tamils, youth in particular, in mass arrests, detention, disappearances and killings to the counter violence of the Tamil militants to control the populations; a fluid and shifting terror was there just below the surface, subtle and covert but an important part of the ecological context. A photographic record of this terror can be seen in the faces of the 'Army I.D. cards' issued to those returning to Jaffna after the Army took control in 1996.

The change in the dynamics and social transformation that arise from ecological transitions or shifts [15] from the civil, peace context to a war context would result in change at cognitive, emotional and social levels. In N.S.L., with the vicious spirals of resource loss, there was regression to constriction of consciousness, narrowing of outlook and world views, and reduced social cohesion with suspicion, mistrust and ennui. Studies of adolescents [12], showed a marked impairment in cognitive functioning. These can be discerned in adults also. Particularly, there was a constriction or narrowing of cognition, thinking in general had become very restrictive, petty, mundane, rigid, fixed on survival and self-interest. A characteristic feature of traumatization is loss of concern about the future, lack of planning (DSM IV). People learned to survive from day to day. A marked preoccupation with death as seen in popular media (death announcements, condolence messages, posters commemorating the dead) and less concern with the future was noticeable. The constriction in cognition and the domination of negative emotions led to stereotypic ideas of other groups, paranoia, hatred and revenge. Self-perpetuating cycles of violence and counter violence, terror and counter terror kept a whole young generation growing up in this atmosphere, and society locked into a mind set of violence and war. Non violent solutions to problems were not seen to work. Well intended peace building programmes failed to break this cycle as the fixated minds of decision makers or leaders were not addressed nor were the fundamental inequities fueling the conflict. An attempt at recovery and reconciliation programmes will have to reverse these pernicious cognitive changes and repair the socioeconomic and political causes.

### Social Justice

Another important casualty in this war has been the implicit faith in the world order and justice in particular. The overriding experience of Tamils has been a discriminatory system and injustice. Those responsible for what may be called war crimes and the worst types of human rights abuses have never been punished. The few cases of massacres, disappearances, torture, rape, custodial killings, mass graves that have been investigated and brought to light have not resulted in justice being done. Impunity prevails. Though perpetrators have been identified, and in some rare cases arrested and court cases instituted, none have been sentenced (the sole exemption being the highly publicized Krishanthy case which was taken up by many women's and other human rights organizations), or punished [82]. The perpetrators are promoted (such as diplomatic posts overseas), or they are transferred elsewhere. In the case of abuse and injustices committed by the Tamil militants, there is no social mechanism for redress; the victims usually have to bear it in silence, a silence that is often individual as well as collective.

### Consequences of collective trauma

The cumulative effect of all these devastating events and ecological contexts on the community can be described as collective trauma. In addition to the sum total of individual traumas, which can in itself be substantial given the widespread nature of the traumatization due to disaster(s), there are impacts at the supra-individual family, community and social levels that produce systemic changes in social dynamics, processes, structures and functioning. In fact, the psychosocial reactions in the individual may have come to be accepted as a normal part of life. Thus being tense, ever vigilant, readily startled, irritable, having nightmares and poor sleep and experiencing multiple somatic complaints would not be considered unusual. But at the community level, manifestations of extreme experiences can, for example, be seen in the prevailing coping strategies. People have learned to survive under extraordinarily stressful conditions. A UNHCR official observed that in Jaffna people have become professional in dealing with complex emergencies from previous experiences. Every family has a bag packed with all the vital items to live rough and essential documents, ready to leave at a moments notice. When displaced to a camp, they are very systematic in getting themselves organized. They immediately find a corner, hang up screens with *sarees*, and start arranging their belongings for a stay. It should be also mentioned they are now very adamant in obtaining, even demanding dry rations and other relief items. This 'previous training' came in handy after the Tsunami, where the displaced from the Tamil community were found to cope much better than their Southern counterparts. Many of the Tamil psychosocial training manuals that had been developed over the years were translated into Sinhalese after the tsunami.

However, some coping strategies that may have had survival value during intense conflict may become maladaptive during reconstruction and peace (see Table 3). For example, the Tamil community had learned to be silent, uninvolved and to stay in the background which would have helped in survival. They have developed a ***deep suspicion ***and ***mistrust***. For example the Tamil people no longer trust the security forces, including the police. Their recent experiences have taught them otherwise. Thus instead of trust, respect for Police, and a belief in their legitimacy; there is fear, even terror. Thus when someone breaks the law, or there is a robbery or some other illegal activity, no Tamil would naturally report it to the Police. A recent example was the UNDP mine awareness programme where the UNDP naively asked people, when they discover a mine, to report it to the local security forces. People protested and the UNDP changed its policy. Similar paranoid attitudes were found after the Sept. 9/11 attack in New York [83]. Trust is the basic binding glue that keeps communities and societies together. Trust in relationships, that they will not be betrayed, that others will fulfill their obligations, responsibilities and undertakings, that their intentions are benign; trust in social structures and institutions, justice, law, values and cultural beliefs, the future and finally a trust in themselves, their family and kin. Trust is gradually destroyed by war. This cohesive force is progressively weakened setting in motion a vicious self fulfilling cycle, spiraling downwards of increasing suspicion and mistrust.

**Table 3:** Consequences of collective trauma

Mistrust
Suspicion
"Conspiracy of Silence"
Brutalization
Deterioration in morals and values
Poor leadership
Dependency
Passiveness
Despair
Superficial and short term goals

People have learned to simply attend to their immediate needs and survive to the next day. Any involvement or participation carried considerable risk, particularly at the frequent changes in those in power. The repeated displacements, disruption of livelihood have made people dependent on handouts and relief rations. Similar to Seligman's '*learned helplessness*' [84], this dependence hampers rehabilitation and development efforts. People have lost their self-reliance, earlier a hallmark of Tamil society. They have lost their motivation for advancement, progress or betterment. There is a general sense of resignation to fate. People no longer feel motivated to work, or better their lots. Many prefer to continue to live in refugee camps refusing resettlement plans. After the recent cessation of hostilities in 2002, there were concerted efforts to resettle displaced families by Governmental and Non Governmental Organizations. But many refused to move. In hindsight, this may have been a wise instinct born of previous experiences (the conflict restarted in 2006 and people were re-displaced once again). But even within refugee camps, people did not show interest in self-help programmes like vocational training and income generating projects. Outside camps, people appear to have resigned themselves to just surviving. Similar to what Lifton [85] found in Hiroshima survivors, "*a pervasive tendency to sluggish despair*....." They seemed to live a half life, as though they were '*walking corpses*' or the *living dead*. Many farmlands remain uncultivated, houses un-repaired.

### Brutalization

Another conspicuous collective phenomenon due to the war has been the brutalization of society. Apart from the militarization of all aspects of life (with the ubiquitous check points, armed men, weapons, checking, barbed wire) and the pervasiveness of the 'gun culture', is the long-term effect on thinking and behaviour patterns. Witnessing the horrifying deaths (including killings) of loved ones, friends or strangers, seeing many mutilated or dismembered bodies, decaying and bloated remains have saturated the consciousness with death as evidenced by drawings, dreams, and poetry. Similarly, watching the destruction of what had been a permanent structure, like a home, or having to abandon ones' home under forced circumstances appeared to result in the perceived collapse of everything secure and strong, particularly in children who lost the hopes for a future With time people have become habituated to such scenes and experiences. In a way they were immunized to the worst aspects of the war, able to carry on nevertheless, attend to immediate survival needs in the midst of the destruction and death, a form of resilience.

One worrying development identified in many community focus group discussions is the anti-social personality development in male adolescents and youth. It was noticeable after the Tsunami, where the adolescents and youth in displaced camps seemed to drift into anti-social groups and activities. Initially, during the Tsunami they had behaved heroically saving many and actively helping in the rescue efforts. In the camps too they were in the forefront in organizing activities, programmes and services. However, many of them had lost a parent or sibling. Grief seemed to soon overwhelm them. Further, in some families the family dynamics soon worked to blaming the youth for some of the deaths, not acting appropriately to save a sibling for example. Thus guilt complicated the grief reactions. Further, no ongoing, constructive activities or programmes were designed and implemented for adolescents and youth. They were left out of the school based programmes or those for children. Unemployed and at a loose ends, they drifted into groups and antisocial activities. Some left to join armed militant groups. Others started abusing alcohol. Alcohol abuse also increased among widowed men and became a major problem in the camps. Attempts by camp and community officials to restrict the availability of alcohol or control its abuse were not successful.

Similar antisocial personality development, particularly in male adolescents and youths, was seen in post war settings (long-term ceasefires, cessation of hostilities). Parents and elders in the community who had traditionally been respected no longer had control. These youths were immersed in an atmosphere of extreme violence. Many had witnessed horrifying deaths of relations, the destruction of their homes and social institutions. They were surrounded by war equipment and paraphernalia. They had personally experienced bombings, shelling, extrajudicial killings and displacement. With no avenues for advancement or hope for the future, knowing only camp life, unemployment, and poverty, adolescents and youths formed into violent groups and criminal gangs. Inter-group rivalry, violence and clashes developed to an alarming degree unseen in conservative Jaffna before.

There were inter-gang conflicts and violent fights, spilling over into their communities. Thefts and other antisocial activities like abduction and sexual assault of females, harassment and abuse became common. Parents, teachers and community members expressed difficulties and fear in handling adolescents and youth. This was the age group, which in normal times would have been involved in constructive social activities, advancing and nurturing society with youthful exuberance and altruism [12,86].

At workshops and meetings with adolescents and youth, it was clear that there was considerable pent up anger and resentment against the military, which was seen as an occupying force, responsible for atrocities and violence. The emotions and hostility were just beneath the surface, when given an opportunity to express and ventilate their feelings within the secure but somewhat suggestive and encouraging atmosphere of the workshops, the aggression came out clearly. When asked to hit out (at objects like a pillow) if they felt like it, invariably the pillow was imagined to be a soldier, and considerable aggression was vented in these group settings. Some discerning workers and INGO's expressed concern at the manipulation of this anger by pro-militant organizations used for recruitment and propaganda purposes.

### Social deterioration

The signs of collective trauma can be discerned in many fundamental social processes. Compared to pre-war times, there seems to be a general ennui. People have left their homes and property in disrepair, not taking the effort to start repairs. In offices and organizations, the work output was reported to have declined considerably. Once a *work ethic *dominated this hard working society, now one often hears the complaint that most people are not inclined to work hard, but merely sign their names in the work register and take the day off for the slightest reason. More effort and interest is seen to be spent on obtaining relief items, rations, incentive payments, risk allowances and such like. At times, difficulties have resulted in people fighting over limited resources or facilities, for example in the queues for rations, or seats on the ship to Trincomalee.

There appears to be a crisis of leadership. People are reluctant to take leadership positions like chairmanships or presidencies (unlike before, there is no active campaigning or canvassing, though there was a brief increase in interest during the ceasefire). The considerable threat to those aspiring to leadership roles, many having been killed or intimidated into subjugation, has meant that few would take the risk nor would their family or community allow it. Most positions go by default. There is a noticeable lack of quality in civil society, partly due to the crippling brain drain, but also due to the devastating effect of the war.

There is also widely reported perception in northern Sri Lanka that there has been a marked deterioration in social values evidenced by changing sexual and social behaviours. Although there has been considerable changes in society due to modernization and globalization, but war and displacement may have accelerated these changes so much so that people attribute the perceived changes to recent events. For example, in the refugee camps in Vavuniya and in general society in northern Sri Lanka, medical personnel report increased unwanted pregnancies, teenage abortions and child sexual abuse This has also been attributed to the reduction of privacy in cases of displacement, to increased alcoholism and to the inability of parents to keep an eye on their children in camps. Robberies of the houses and property of those displaced is commonplace. Widespread looting by the public (indulged in even by socially respectable teachers and others highly placed members of society) was seen for example in the wake of many army operations such as after the Indian intervention in 1987 and in Thenmarachi in April 1996 when the army allowed people to return to Jaffna. There is currently a dramatic increase in the number of child abuse cases, including sexual abuse, being reported in Jaffna to the District Child Protection Committee showing an increase during periods of war and a decrease during the ceasefire[87]. A recent survey found that 96% of children had experienced violence at home, with 52% indicating more than 5 violent events, and that most violence was ongoing [88].

This apparent increase in child abuse could be due to increased awareness of the problem (child abuse has always being there in our society, as has teenage pregnancy, but it is only now coming to light) but it is also due to the new stresses due to the war. Many families are displaced from their familiar surroundings and natural habitat where there was the support and protection of others from the village, their extended family and friends. They now have to live in crowded camps or accommodation in strange and new places. Parents too have to go out to attend to various urgent requirements like obtaining relief, rations, and meeting authorities. Some families are separated without their men. In some families, the father and/or mother has started another relationship leaving the children vulnerable to abuse.

There has also been noticeable increase in violence against women as evidenced by the number of battered women seen through the *Kavasam *(Women protection) programme at General Hospital Jaffna [62].

An example of deterioration in morale and loss of sensitivity in being human is from the health sector where, until recently, there was a spirit of service [89]. Medical staff would stay with their patients, sometimes sacrificing their own well-being for the interests of the patient. The collective experience of what happened at General Hospital, Jaffna on Theepavali day in 1987 has made most staff lose their altruism. During that fateful period, staff decided to stick to the hospital and patients despite considerable risk. When the Indian Army entered and massacred both patients and staff, this last bit of service ideal died, too. Staff now look after their self-interest first. At the slightest hint of trouble, they abandon the hospital, their responsibilities, and patients, as happened during the intensification of the conflict in May 2000.

### Child soldiers

**Figure 3:**
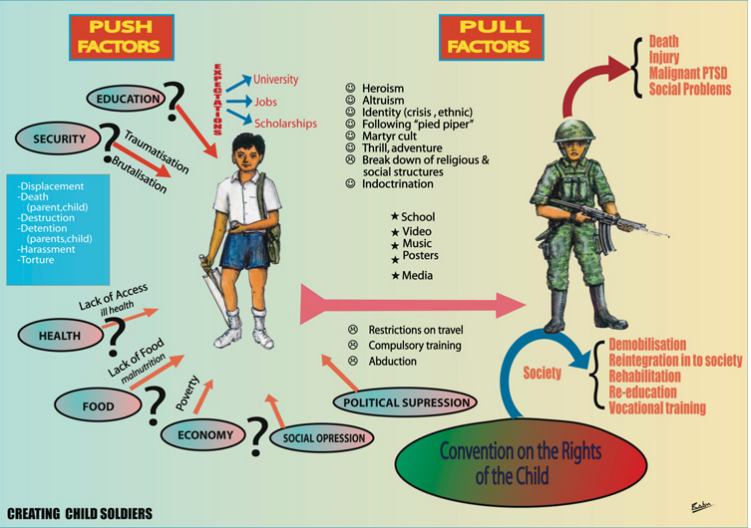
Creating child soldiers – push and pull factors.

It is in this context of psychological, social, economical and political deprivation that the phenomenon of child soldiers becomes possible. A whole generation of Tamil children has been lost, who in the normal run of things, should become the energetic developers of their society. It may not be enough to just merely condemn the recruitment of children, but to ask the deeper question, "*Why do children join the militants*"? It is as important to understand the context under which children become soldiers and work to improve these conditions if this practice is to be effectively prevented [90]. They can be divided into push and pull factors (see Fig. 3). Some of the push factors include death of one or both parents or relations; separations; destruction of home and belongings; displacement; lack of adequate or nutritious food; ill health; economic difficulties, poverty; lack of access to education; not having any avenues for future employment and advancement; social and political oppression of the group, and facing harassment, abductions, detention and death. Opportunities for and access to further education, sports, foreign scholarships or jobs in the state sector have been progressively restricted by successive Sinhalese-dominated governments, despite the lip service paid to maintaining ethnic ratios. There is an alarming drop out rates and irregular attendance in schools in the Jaffna District, becoming the highest in the island [91] that had traditionally given pre-eminence to education. Ironically the Tamil rebellion that started out as a protest against standardization, a strategy by the state to restrict Tamil admission to Universities on merit, has deteriorated with the war to Jaffna now having to claim disadvantaged area status with guaranteed admissions.

As described the greatest impact of the structural violence and oppression is on the younger generation. A more permanent solution to the phenomena of child soldiers would be to bring pressure on the state to dismantle the socioeconomic and political oppression the children face to prevent them becoming soldiers.

On the other hand, examining the pull factors, it becomes clear that the LTTE and more recently, state backed paramilitary groups have turned to children and females to do their fighting as the older males are no longer joining. The older youths have matured enough to see through the propaganda. Children because of their age, immaturity, curiosity and love for adventure still remain susceptible to 'Pied Piper" enticements through a variety of psychological methods. Recruiters have used public displays of war paraphernalia, funerals and posters of fallen heroes, speeches and videos, particularly shown in schools and temple festivals; heroic songs and stories to cleverly draw out feelings of patriotism. The very strict restrictions on leaving LTTE controlled areas, particularly in the recruitable age group, both ensures that there is a continuing supply of fighters and creating a feeling of being trapped and powerless in potential recruits. In addition the aforementioned actions of the Sri Lankan forces all have created a milieu where children are psychologically compelled to join. When these have failed more coercive means, including threats to parents, abductions and press ganging have been employed. The LTTE has introduced compulsory military type training for all eligible ages in areas under their influence. This instills a military thinking and lead to more joining.

In this context, it is easy to understand why joining a powerful group can become an alluring alternative. Those responsible for the recruitment, training and deployment of child soldiers as well as those perpetuating the socio-economic and politically oppressive conditions should be considered as war criminals while the child soldiers themselves should not be treated as criminals or juvenile delinquents as they are now. They are but victims of the system, the ecological context and should be offered appropriate psychological, socio-economic and educational opportunities for rehabilitation as is being attempted currently by the UNICEF. The tragic occurrence at Bindunewava where captured child soldiers were later massacred is a poignant reminder how hypocritical towards the actual welfare of (Tamil) children the state really is.

In these circumstances of recruitment of children, Tamil sociocultural and religious institutions have failed to protest. Apart from functioning as apologists, no Tamil leaders have not dared to comment, let alone condemn. This paralysis may be partly due to the severe human rights violations that have been perpetrated by the Sri Lankan State, and partly because of the general social deterioration caused by the war. But it is also the silence of a community under totalitarian control by the Tamil militants. Only a few affected parents have made muted protests. Thus the Tamil militants have been allowed to function freely within society to attract and pull children through their propaganda and psychological pressure in the vacuum left by the abdication of social institutions.

Although not the focus of this paper, there were some positive, constructive changes in society that could be observed which could be useful in attempts in designing programmes to address the collective trauma. There were the emergence of new community organizations, particularly with active female participation, enhanced female role and leadership [62], breakdown in caste barriers and hierarchical structures [92] and a decrease in suicide rates during active periods of conflict [93]. Together with the resilience gained from surviving decades of war and the potential post traumatic growth, these positive developments need to be utilized in the recovery process and start the resource gain cycle [16].

## Collective strategies

Traditionally, disaster interventions have been categorized into rescue, relief, rehabilitation, reconstruction and development depending primarily on time course after the disaster. Apart from attending to the immediate basic needs and other acute problems in the rescue and relief phases after a major disaster, rehabilitation, reconstruction and development strategies need to include collective level interventions [23-25]. Indeed, it has been our experience that many long-term programmes do not reap the expected benefits if they do not take into consideration collective issues and ways of addressing them. In fact, many individual oriented interventions appear to fair much better when undertaken within an overall collective level design.

### Models of mental health interventions

**Figure 4:**
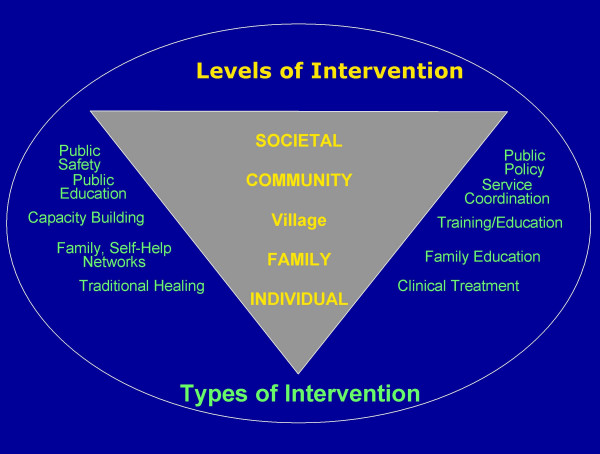
Conceptual model for psychosocial interventions in social and humanitarian crises [1].

One approach is to use the WHO definition of health already mentioned to address the physical and psychosocial needs of the survivors through physical and psychosocial interventions (Table 1). Another more comprehensive and useful conceptual model (Fig. 4) for psychosocial and mental health interventions is an inverted pyramid with five overlapping and interrelated levels of intervention prepared for UN and other Disaster workers by the United Nations and International Society for Traumatic Stress Studies [2]. As shown in Figure 3, at the top of the pyramid are societal interventions designed for an entire population, such as laws, public safety, public policy, programmes, social justice, and a free press. Descending the pyramid, interventions target progressively smaller groups of people. The next two layers concerns community level interventions which include public education, support for community leaders, development of social infrastructure, empowerment, cultural rituals and ceremonies, service coordination, training and education of grass root workers, and capacity building. The fourth layer is family interventions that focus both on the individual within a family context and on strategies to promote wellbeing of the family as a whole. The bottom layer of the pyramid concerns interventions designed for the individual with psychological symptoms or psychiatric disorders. These include psychiatric, medical and psychological treatments which are the most expensive and labour intensive approaches that require highly trained professional staff. Therefore, they should be reserved, particularly in the poor, developing world settings, for the small minority of individuals who cannot benefit from the larger scale interventions at higher levels of the pyramid. Theoretical, practical, empirical and treatment considerations suggests a holistic, integrated, multiple levels of interventions, an expanded psychosocial synthetic approach [93].

### Community approaches

The wide spread problem of collective traumatization and '*loss of communality*' following disasters is best approached through community level interventions. Community based approaches will enable one to reach a larger target population as well as undertake preventive and promotional public mental health activities at the same time. In these circumstances it may be more meaningful to look at how the community as a whole has responded, how the community coped, and what we can do at the collective level. For example, it may be more beneficial to consider strengthening and rebuilding the family and village structures, as well as finding a common meaning for the immense suffering than to treat only individual traumatization. For this purpose, the protocol developed by the Transcultural Psychosocial Organization, a WHO collaborating centre, working around the world to relieve the psychosocial problems of people affected by internal conflict and war [95] was very effectively adapted to the situation in Cambodia [96] and in northern Sri Lanka [89]. The main principles of community level approaches (Table 4) are to empower the community to look after their own problems by not only through psychoeducation to transfer basic psychosocial knowledge and skills but also through encouragement, support, affirmation and re-establishing community processes, traditional practices, rituals, resources and relationships.

**Table 4:** Community approaches

Community Approaches	
Awareness	
Training of community workers	
Public mental health promotive activities	
Encourage indigenous coping strategies	
Cultural rituals and ceremonies	
Community interventions	
	- Family
	- Groups
	- Expressive methods
	- Rehabilitation
Prevention	

### General awareness

Basic information about what has happened, what to do and not to do and where help can be obtained were done through the media, pamphlets and popular lectures. One such popular programme was carried out through the Extra Mural Studies unit of the University of Jaffna where batches of around 100 participants were taken through a basic introduction to psychosocial issues over a five week period. Five such courses were completed. Another major psychoeducational effort was carried out immediately after the Tsunami by Mental Health Task Force in Jaffna using the media, pamphlets and lectures. The Mental Health Task Force (MHTF) was formed spontaneously after the Tsunami by most of the organizations involved in psychosocial work in the Jaffna peninsula which then met regularly and attempted to organize and coordinate the psychosocial work after the tsunami. MHTF now continues as the Psychosocial Coordination Forum under the Deputy Director of Health Services, Jaffna. After the Tsunami the MHTF carried out 18 awareness programmes for 568 relief workers and others. Similarly the committees involved in Child abuse and Domestic violence carried out considerable to public education programmes from 1999 which resulted in increased numbers being reported (see above) and being helped.

### Training

Training of grass root community level workers in basic mental health knowledge and skills is the easiest way of reaching a large population [97]. They in turn would increase general awareness and disseminate the knowledge as well as do preventive and promotional work. Thus there would be a multiplication effect where the information would spread to the general population. The majority of minor mental health problems following disasters could be managed by community level workers and others referred to the appropriate level. A referral system where more severe problems are referred for more specialized treatment was established (Fig. 2). Primary Health Workers including doctors, medical assistants, nurses, Family Health Workers; school teachers; village resources like the village headman, elders, traditional healers, priests, monks and nuns; Governmental, Non Governmental Organization (NGO), volunteer relief and refugee camp workers were community level workers who were trained. A manual based on the WHO/UNHCR [98] booklet, "*Mental Health of Refugees"*, was adapted to the cultural context for this purpose [60]. A Training of Trainers (TOT's) in community mental health using this manual was done under a UNICEF programme. They in turn trained a variety of community level workers from the North and East and have followed them up regularly in the field (for example, after the Tsunami, there were 36 different training programmes for 11 agencies with a total of 732 participants over a 9 month period in 2005).

A systematic training programme for teachers in basic mental health for a period of six months with assessments and follow up were carried out with the German supported GTZ-BECAre programmes and Ministry of Education, University of Jaffna and Shantiaham, a local NGO working in the psychosocial field. Altogether around 151 primary school teachers from all three communities selected from the North and East were trained in the period 2002–2005 using a manual, Child Mental Health, developed locally for this purpose [99]. They then received further extensive training in Narrative Exposure Therapy (NET) [100] by a German team for the University of Konstanz (Vivo) and a manual for NET made available in Tamil. An impact study [101] found that there had been considerable change in the attitude and skills of the teachers who now interacted more with their students, parents and colleagues, avoided punishment and created a more caring, participatory and democratic class atmosphere apart from carrying out a variety of psychosocial interventions for affected students. They were then involved in the training of around 1030 'Befriender' teachers from the same region in simple psychosocial issues over a 5 day course using a manual, '*Joyful Living*', prepared for this purpose [102]. Regular follow-up and supervision locally was carried out. Yet, the whole programme faltered after the German GTZ changed its programme focus from 2005. One advantage of all this training that had been done during the war period became apparent when the tsunami struck in 2004. The trained teachers were mobilized to assist the affected students throughout the North East. The benefits of this programme became evident nationally and the manual was translated into Sinhalese. Copies in Sinhala and Tamil were made available in all the schools in the Island through the efforts of PLAN International, and psychosocial training for teachers in the Tsunami affected areas was done.

### Traditional coping strategies

Indigenous coping strategies that had helped the local population to survive were encouraged. Culturally mediated protective factors like rituals and ceremonies were promoted and arranged. For example funerals and anniversaries, were very powerful ways to help in grieving and finding comfort. They were a source of strength, support and meaning.

Teaching of the culturally appropriate relaxation exercises to large groups in the community and to students in schools was carried out as both preventive and promotive mental health. The benefits of these originally spiritual practices were not confined to producing relaxation. When methods are culturally familiar, they tap into past childhood, community and religious roots and thus release a rich source of associations that can be helpful in the healing process. It became clear that traditionally relaxation methods exemplify a holistic approach working at the physical, mental, social and spiritual levels; preventing, maintaining, promoting well being as well as being therapeutic when needed [59]. We found that people naturally turned to traditional practices when under stress and found relief in them. For example, in Sri Lanka we found religious practices such as *ana pana sati*, repetition of meaningful phrases such as *Buddham saranam gacchami, mindfulness*, and *vipassana *meditation among Buddhists; *rosary *or telling *prayer beads *and *contemplation *among Catholic Christians; *thikir *among Muslims; and *Japa mala*, repetition of a *mantra *such as *om, shanthi asanam, yoga, pranayamam and meditation *among Hindus were powerful methods known to the people and priests.

### Community interventions

#### Group support

The formation of groups for survivors, affected families, widows, ex-detainees, torture survivors, landmine victims and other groupings can be very helpful. A therapist would facilitate the interactions within these groups, which in time should develop its own healing and caring processes. A widows' support programme funded by the Government of Finland was started in a particularly badly affected fishing village, Chavatkaddu, which had a high number of war widows. The widows met as a group to support each other, exchange their stories and recount experiences. Coping strategies used by one person could be tried by another. The widows were able to organize themselves into a powerful group to overcome stigma and exclusion in their village, undertake joint economic ventures, obtain relief and rehabilitation projects, arrange for education and tuition for their children, go on tours together, celebrate religious and cultural festivals and observe death anniversaries. In time they were able to expand their programmes to other villages, training their counterparts and helping them to organize themselves.

#### Family support

In our cultures where the family bonds are very strong, the family is an essential resource that can be used for healing. Efforts were taken to keep the family together and functioning. For example, attempts to separate orphans into institutions were resisted through the District Child Protection Committee (DCPC), and efforts were made to keep and support the child with their near relations. In time this principle was accepted and adopted by the National Child Protection Authority after the issue arose after the Tsunami. Other agencies like the ICRC were contacted to trace missing relations and unite the family. Family cohesion was strengthened. The principles of family dynamics were used to facilitate supportive and healing relationships while counteracting damaging and maladaptive interactions. Communication of individual problems leading to an awareness of each other, one's role and encouragement towards mutually interdependent functioning were used to build up family unity. Traditional roles had to be re-established. These considerations were used for the extended family as well. When individuals, particularly children, presented with problems due to pathological family dynamics, it was the family that had to be managed if the individual was to recover [12].

#### Village level psychosocial interventions

Badly affected villages by the war and later, by the tsunami, were selected for psychosocial work. The steps in the intervention programme in each village are given in Table 5 and the psychosocial interventions in Table 6. Although broken up as steps, they were in reality all ongoing processes, with the monitoring evaluation and assessment fedback into the programme to adjust and modify the design and implementation based on lessons learnt and contextual factors. For example, after the tsunami the interventions had to be made more appropriate for the male widows or some areas or aspects of programmes changed due to developing security concerns.

**Table 5:** Steps for community based psychosocial work

**Step**	**Title**	**Description**
**1**	**Village assessment**	information from GA, DS, GS, psychiatrists, counsellors, psychiatric social workers, health workers, psychosocial workers and the representatives of the organisations from that region (statistical data)
**2**	**Selection of the villages**	Based on Poverty, Affected by war (death, injured, missing), Displacement, Resettlement, Socioeconomic problems, Domestic violence, Alcohol and drug abuse, Natural Disasters (e.g. Tsunami), Child abuse
**3**	**Obtaining permission**	Permission will be obtained from the government authorities to work in the selected villages. E.g. Divisional Secretariat, Grama Sevaka Officers
**4**	**Integration with people**	introduction to Villages about the worker, organisation and intention of the activities
**5**	**Meeting with resources**	discuss with the most important resources from the village to get their whole support
**6**	**Cross walk**	Looking around all the nook and corners of the village
**7**	**Learning about the society **	Getting to know the language, culture, traditions, rituals and occupations with the help of the important resources who are living in that area
**8**	**Data collection & documentation**	Basic Demographic data about the village
**9**	**Social Mapping**	Ecosystem of society, places where collective trauma occurred, the house of the community leader, temples, CBO's, Traditional leaders
**10**	**Identifying and analysing the problems**	Through Key informant interviews, Focus group interviews
**11**	**Planning**	Based on the abovementioned identified problems and their priorities
**12**	**Community meetings and creating awareness**	First with the key resources, then for the whole community, explain about plans, benefits, psychosocial wellbeing, prioritised problems of the community. E.g. Alcohol awareness, awareness of child abuse, domestic violence etc.
**13**	**Selection of the Core Group (CG)**	A local group which is made up of teachers, university students, farmers, villagers and contains 15 – 20 people with gender balance
**14**	**Core Group training**	Focused on psychosocial well being and psychosocial problems in the village level, referral and networking.
**15**	**Working with Core Group**	Trainer works with CG in Social mobilization, community awareness, children group activities, Identification of psychosocial problems in the community, psychosocial interventions for individuals, families and community, facilitate women groups, following up the past cases, doing referral and network for new cases etc.
**16**	**Psychosocial Interventions**	See Table 6
**17**	**Core Group follow up**	After handing over the village to the Core Group, they will continue work in village. Supervision and further training in particular.
**18**	**Referral**	Problems which the CG is unable to handle would be referred to mental health professionals
**19**	**Networking**	Working with GO's and NGO's for socio-economic and other needs
**20**	**Monitoring and evaluation**	Feedback into modify Planning stage and programme implementation. Design of new programmes.

-Vijayashankar, PSW Trainer, Shantiaham

Consequent to the Tsunami of Dec. 2004, the WHO developed a strategy of training Community Support Officers (CSO's) to address the mental health needs of the affected families throughout the areas devastated by the Tsunami in Northern Sri Lanka. In pursuance of this aim, CSO's were selected from Primary Health and other community level workers from Vadamarachy East, Maruthenkerny, Killinochi and Mullaithivu (see Fig. 1) and given a basic training in basic mental health and psychosocial work. They then visited each family affected by the Tsunami and identified families and individuals needing help. They attended to simple problems using a variety of psychosocial interventions (Table 6) and referred more difficult cases to the local mental health clinic. Community interventions were also carried out. There was regular supervision and follow-up.

**Table 6:** Essential psychosocial interventions

**Individual**	**Family**	**Social**
Case identification	Psycho education	Awareness
Psycho education	Family counselling	Training
Counselling	Strengthening the family dynamics e.g. Talking and eating together	Intervention for special groups (children, widows, widower, youths etc)
Other Psychotherapy	Family Reunification	Encourage to do religious and ritual activities
Yoga and Relaxation	Social support	Encourage to do cultural activities
Family & social support	Capacity Building and Income generation	Forming and reactivating CBO's.
Referral and net work	Follow up	Re-establishing relationships, social networks
Capacity Building and Income generation		Network with other NGOs
Rehabilitation		Encouraging networking with other communities.
Follow up		Follow up

-Vijayashankar, PSW Trainer, Shantiaham

#### Expressive methods

Structured play activity for children were arranged to enhance recovery in post-disaster situations. Children's time was usefully structured and physical energy channelled in a healthy manner. More important, it helped organize community or camp activity, build friendships and peer relationships and give an opportunity for the child to bring out his or her emotions and creative impulses.

In a collaborative programme involving the Ministry of Education, Danish and Sri Lanka Red Cross and Shantiaham, 150 teachers and 118 volunteers were trained during 2004–5, to implement structured activity in badly affected schools in the Jaffna peninsula using a manual, "*We are little children*", prepared for this purpose. The aim was to promote psychosocial well being through play, art, dancing, stories, yoga, creative and emotional expression and involvement of the parents. A total of 2,800 children from 19 schools and 4,000 parents were expected to take part in the activities in 2005 and 2006. With the acceptance of this methodology by the National Institute of Education in the aftermath of the tsunami, the manual was then translated into Sinhalese and distributed to all schools nationally. However, the whole programme collapsed when the Danish Red Cross decided to pull out of Jaffna after the resurgence of the war in 2005–6. A similar Class Based Intervention (CBI) developed by Robert Macy and colleagues at the Centre for Trauma Psychology, Boston using structured play activityover 5 weeks for15 sessions where 1455 students from 30 schools underwent this programme from 2004 to 2007.

Psychological and socio-dramas can go a long way in creating awareness about trauma among the public and help traumatized individuals to ventilate their emotions or seek treatment. A few such dramas were produced in the Northern Sri Lanka. There were also attempts in the north to use drama and street plays as well as art to work with children affected by the tsunami [103].

#### Rehabilitation

Attempts were made to rebuild social networks and sense of community by encouraging and facilitating formation of organizations, rural societies, schools and the like. Social mobilization was promoted by tapping local resources, re-establishing traditional relationships, practices and engendering leadership from among the community. For example, a local widow from Chavatkadu who was trained as a psychosocial worker became the head of the their widow's organization, *Tharaka*, and was later nominated for the Nobel prize in 1985 with 1000 other leading women worldwide for their services by an international women's group [104].

**Figure 5:**
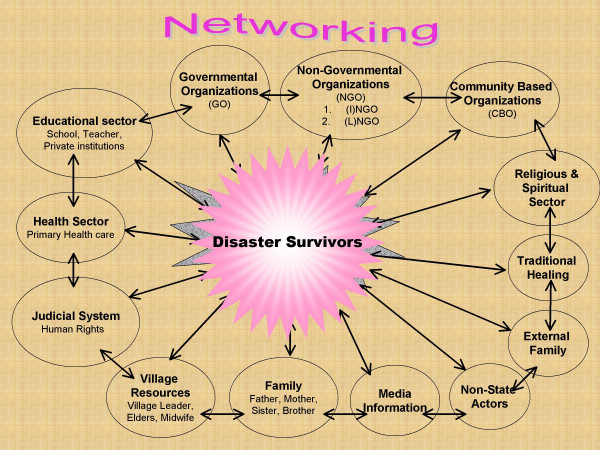
Networking – working with governmental and non-governmental organizations.

Rehabilitation programmes were encouraged to include education, vocational training, income generating projects, loans and housing that is tailored to the needs of the survivors and post disaster situation. Close liaison, co-operation and networking with governmental and NGO's involved in relief, rehabilitation, reconstruction and development work was attempted (Fig. 5). The network was used to refer needy survivors for relief, socio-economic rehabilitation, legal aid, shelter, nutrition, water and sanitation, human rights, protection and other assistance (For recommended guidelines see IASC [25]).

A holistic integrated approach was advocated with Governmental and Non Governmental aid agencies emphasising the need for planning that included due consideration for the psychological processes that promote individual, family and social healing, recovery and integration. Such programmes were encouraged to take into account the wishes of the local population concerned, that they be given an active and deciding role rather than a dependent, 'victims' role, as it promotes their overall sense of participation and thus their eventual psychological recovery. To avoid this, emergent self-help groups and local leadership were encouraged to resume traditional and habitual patterns of behaviour, re-establish social networks and community functioning at the grass root level [105]. The local skills and resources were tapped and utilized, which gave the community a sense of accomplishment and fulfilment in the recovery process. Provision for the non-partisan cultural working through of the shared traumatic experience in the form of periodic reminders of the loss and reiteration of its meaning, and of the heroism of those who suffered expressed in media, arts, public works, monuments, and occasions of public mourning were encouraged as they have been found to be useful in post disaster situations [106]. For example, after the tsunami, affected schools were encouraged to have regular ceremonies to commemorate those who died, to have pictures, flowers and candles for the students who had died and support was sought for communities which sought to build memorial structures at sites of mass burials where public gatherings, meetings and religious ceremonies will allow for communal release of feeling, review and coming to terms with the **collective trauma**; socially define and interpret their experiences, as well as re-establishing social relationships and planning for the future. It has been found that sites where mass trauma has occurred become sacred, imbibed with community meaning [107]. However this was not always allowed in the charged political situation that prevailed in these areas. For example, in the coastal villages of Maruthernkerny where 901 people out of a local population of 16,153 died due to the tsunami, there had been hasty burials in mass graves. As a method of consoling the traumatic grief of the surviving family members, attempts to build memorials at these sites where people could visit, grieve, remember and perform rituals were consistently blocked.

In the polarized and totalitarian political situation with competing loci of power and parallel governments, community organization, mobilization and empowering was only allowed to proceed up to a point. When the organization or mobilization started to become effective, political forces took over, infiltrated or interfered in the process. In the Tamil areas no independent large scale organization or activity was allowed. Perhaps it was taken as a challenge to the existing social arrangements, control, dispensation, and loyalties. More than the state, it was the Tamil militants who were very sensitive to such activity and became very efficient in organizing and implementing relief, rehabilitation and development programmes. There was a need for the political organization to be seen as the one doing the construction, channelling the aid, receiving credit and legitimacy. Co-operation with the political forces on the ground was the only realistic option available and the route taken by the GO's, NGO's and INGO's. But, ultimately political and military priorities such as recruitment took precedence. Independent social activity, particularly beyond a critical, effective level carried an intrinsic risk. Thus, in such situations, workers need in addition to cultural competency, political competency as well. Every act takes on a political significance. Tragically, even the post Tsunami rehabilitation process and programmes became political. Unlike in Aceh, an opportunity for addressing a humanitarian need in an equitable, neutral way to build trust and faith in peaceful rehabilitation to bring warring parties together was lost.

#### Prevention, policy, planning

Tragically, much of the deaths and destruction caused by natural disasters can be avoided. This is even truer for the man made disaster that is war. Ahimsa, peaceful coexistence, conflict resolution, reconciliation and other values were advocated and passed on wherever possible. However, it is sobering that despite all this effort and training, the country has once again returned to full scale hostilities since December of 2005.

Just as the consequences of disasters must be addressed on both community and family levels, so must plans for preventing or mitigating their impact. In fact, there should be plans at the local, district, provincial, national, regional and international levels for disaster preparedness and emergency response because many disasters affect multiple communities, nations or regions. Such plans are typically formulated by committees at the appropriate level and may involve collaborative efforts between formal emergency management agencies, public health and other agencies, and citizen groups. Small steps were taken in formulating disaster plans.

Another area of intervention was at the national, regional and district levels by influencing policy making, rehabilitation and international aid programmes. Memberships at various committees at the local, district, regional and national levels provided the opportunity to make some contribution towards prevention and alleviation of the effects of individual and collective trauma. The problems of putting psychosocial and mental health concerns on the agenda and the general stigma associated with mental health were quite evident at these committees. Although there was wide acceptance of psychosocial problems due to the Tsunami and there were considerable effort to address these by the state, militants, NGO and INGO sectors; the state is still to accept or take responsibility for the psychosocial problems arising from the war. As such it was left to INGO and local NGO's to carry out psychosocial interventions and programmes. Unfortunately many of these internationally supported programmes and structures tended to collapse when the funding stopped or INGO's pulled out. The local partners and government were not able to maintain the momentum. This raises the question of long term sustainability. The national discrimination, inequity in distribution of resources and programmes, and exclusion of the North and East continued to be insurmountable hurdles [108].

## Conclusion

The effects of disasters, particularly massive, chronic traumas go beyond the individual to the family, community and wider society. Social processes, dynamics and functioning can be changed fundamentally by disasters. In the aftermath of war some of the community level changes included mistrust, suspicion, silence, brutalization, deterioration in morals and values, poor leadership, dependency, passiveness and despair. It maybe important to recognize the manifestations of collective trauma, so that effective interventions that is effective at the community level can be used in these complex situations. Community approaches that were found useful in rebuilding communities in Northern Sri Lanka were creating public awareness, training of grass root workers, encouraging traditional practices and rituals, promoting positive family and community relationships and processes, rehabilitation and networking with other organizations.

### Limitations

This was only a phenomenological study of collective trauma. There are obvious limitations to dispassionate scientific study due to the ongoing war situation. The case for collective trauma was merely described, no random controlled trials were conducted nor the outcome of the community interventions collected in a systemic way. The evidence base for the efficacy of psychosocial interventions in a post disaster situation is still lacking and only guidelines have emerged from humanitarian work carried out so far [25]. Operational criteria for the syndrome of collective trauma and the evidence for best practice in these complex situations will need to be established through further studies.

## Abbreviations

APA – American Psychiatric Association

BECAre – Basic Education for Children in Disadvantaged Areas

CBO – Community Based Organization

CBT – Cognitive Behavioural Therapy

CSO – Community Support Office

DSM – Diagnostic and Statistical Manual

G.A. – Government Agent (District Administrator)

G.O. – Governmental Organization

G.S. – Gramma Sevaka (Village Headman)

GTZ – German Technical Cooperation

IASC – Inter Agency Standing Committee

INGO – International Non-Governmental Organization

LTTE – Liberation Tigers of Tamil Eelam (dominant Tamil militant group)

N.S.L – Northern Sri Lanka

NGO – Non-Governmental Organization

PTSD – Posttraumatic Stress Disorder

SCF – Save the Children Fund

SSRI – Selective Serotonin Reuptake Inhibitor (Anti depressant)

UNDP – United Nations Development Program

UNHCR – United Nations High Commission for Refugees

UNICEF – United Nations International Children Emergency Fund

WHO – World Health Organization

## Competing interests

In terms of conflict of interest, the author subscribes to a pacifist persuasion, contrasting with the militant/military fervour of the authorities in power. This has added a considerable targeted risk factor, particularly in Northern Sri Lanka, apart from the general uncertainties (terror) of these contexts. The author has been the originator and organizer of various social and humanistic programmes in the region including child rights, women affected by domestic violence, human rights and traditional yoga. This has involved considerable public advocacy and campaigns, the tone of which colours much of the author's writings. The author believes with many other health workers [3,28,29] that there can be no neutral position in these extreme situations.

## Authors' contributions

The author was directly involved in all aspects of this study from the initial participatory observation, analysis and documentation. He was either directly responsible for the specific studies described or functioned in an advisory or supervisory capacity. The author takes responsibility for the conclusions and views expressed here.
